# Evolution of root plasticity responses to variation in soil nutrient distribution and concentration

**DOI:** 10.1111/j.1752-4571.2012.00263.x

**Published:** 2012-12

**Authors:** Judah D Grossman, Kevin J Rice

**Affiliations:** Department of Plant Sciences, University of CaliforniaDavis, CA, USA

**Keywords:** artificial selection, barley, evolution of plasticity, *Hordeum spontaneum*, *Hordeum vulgare*, plant domestication

## Abstract

Root plasticity, a trait that can respond to selective pressure, may help plants forage for nutrients in heterogeneous soils. Agricultural breeding programs have artificially selected for increased yield under comparatively homogeneous soil conditions, potentially decreasing the capacity for plasticity in crop plants like barley (*Hordeum vulgare*). However, the effects of domestication on the evolution of root plasticity are essentially unknown. Using a split container approach, we examined the differences in root plasticity among three domestication levels of barley germplasm (wild, landrace, and cultivar) grown under different concentrations and distribution patterns of soil nutrients. Domestication level, nutrient concentration, and nutrient distribution interactively affected average root diameter; differential root allocation (within-plant plasticity) was greatest in wild barley (*Hordeum spontaneum*), especially under low nutrient levels. Correlations of within-plant root plasticity and plant size were most pronounced in modern cultivars under low-nutrient conditions. Barley plants invested more resources to root systems when grown in low-nutrient soils and allocated more roots to higher-nutrient locations. Root plasticity in barley is scale dependent and varies with domestication level. Although wild barley harbors a greater capacity for within-plant root plasticity than domesticated barley, cultivars exhibited the greatest capacity to translate within-plant plasticity into increased plant size.

## Introduction

Phenotypic plasticity, or the ability of a given genotype to generate a range of phenotypes under different environmental conditions, is a trait that can vary genetically, be adaptive, and evolve in response to selection (Miner et al. 2005). Many plants exhibit root plasticity, whereby plants proliferate roots in fertile microsites and increase ion uptake of specific roots (Fransen et al. 2001; Adams et al. 2002; Hodge 2004). Root plasticity is potentially a heritable trait (Basak and Chaudhuri 1967; Fitz Gerald et al. 2005) and can be an important aspect of a plant’s ability to forage for soil nutrients that often occur in heterogeneous patches. However, root plasticity may not always be beneficial because root growth and maintenance also represent costs to the plant (Rajaniemi 2007).

Although plant morphology and phenology can change substantially during domestication (Vaughan et al. 2007), it is unclear whether plasticity in crop plants has also evolved in response to artificial selection. Studies of biomass allocation and seminal root morphology have documented the differences between domesticated barley cultivars (*Hordeum vulgare* L.) and wild forms of barley, *Hordeum spontaneum* C. Koch (Grando and Ceccarelli 1995; Wacker et al. 2002). Furthermore, genetic diversity in wild barley is greater than in cultivars, suggesting that selection during the domestication process may have had a canalizing effect (Maroof et al. 1995; Varshney et al. 2007). Reduction in genetic diversity might lead to lower capacity for plasticity in general, and root plasticity in particular, but we are not aware of any studies that have specifically examined the effects of domestication on root plasticity.

Conventional agricultural practices have a tendency to increase soil nutrient levels and homogenize soil nutrient distribution (Jackson and Bloom 1990; Jackson and Caldwell 1993; Paz-Gonzalez et al. 2000; Bennett et al. 2005). Regularly spaced roots are most efficient in homogeneous soils because they minimize overlap between root uptake zones (Rubio et al. 2001). Over the last century, modern cereal cultivars have been selected on land subject to conventional soil management. Homogenization of the distribution of soil nutrients, coupled with agricultural breeding programs that typically reduce genetic variation within crop species, suggests that domesticated accessions of cereal crops should exhibit a reduced capacity for root plasticity in response to soil heterogeneity compared with wild forms.

Root plasticity can be defined at two spatial scales: (i) the level at which an individual plant’s root system encounters multiple soil conditions (i.e., within-plant plasticity) and (ii) the level at which different plants of the same genotype or accession are exposed to different soil conditions (i.e., among-plant plasticity). It is important to distinguish between among- versus within-plant root plasticity because different scales of soil nutrient heterogeneity can stimulate different responses in root allocation. For example, plants may vary total allocation to roots in response to different average soil nutrient levels (among-plant plasticity), while at the same time, preferentially proliferating roots within high-nutrient microsites (within-plant plasticity) (Reynolds and D’Antonio 1996; He et al. 2003).

Cereal crops are good model systems for the study of root plasticity because crops have been subject to artificial selection for increased yield on cultivated soils, and the identification of germplasm harboring advantageous root plasticity traits might have practical applications in agricultural breeding programs. Barley is a particularly good candidate for the study of root plasticity because barley roots have been shown to proliferate in rooting media with elevated phosphate, ammonium, and nitrate (Drew 1975). In addition, barley has been under continued domestication for the last 10 000 years, resulting in two groups of domesticated forms in barley (traditional landraces and modern cultivars) that are distinct from each other and from wild barley (Badr et al. 2000).

In the early 20th century, accessions of barley termed ‘old’ or ‘traditional’ landraces were collected and stored in gene banks. Since that time, barley-breeding programs have produced modern cultivars, although wild barley continues to persist on uncultivated lands. One reason we chose barley as a model system was because germplasm samples were readily available for these three distinct levels of domestication: wild forms, landraces, and modern cultivars (Parzies et al. 2000; Wacker et al. 2002). Seed selection at the farm level and modern agricultural breeding yield similar results, suggesting that landraces and modern cultivars share a similar domestication process that distinguishes them from wild barley (Ceccarelli et al. 2000).

In this study, we experimentally manipulated soil nutrient level and distribution to examine root plasticity at two spatial scales and to evaluate whether barley has evolved changes in root plasticity in response to varying levels of domestication.

## Materials and methods

### Growing conditions

Barley seeds were planted in round pots (934 cu cm) on open-air, full-sun bench tops in Davis, California, USA (38°33′N, 121°47′W) on April 20, 2007, and harvested on May 18, 2007. Average environmental conditions during this period, measured by California Irrigation Management Information System No. Six in Davis, California, included 26.2°C maximum air temperature, 8.5°C minimum air temperature, 244.8 Watts m^−2^ solar radiation, 83% maximum relative humidity, and 27% minimum relative humidity. Pots were watered to field capacity with tap water on an as-needed basis; all pots were watered at the same time. To avoid placement bias, treatment replicates were ordered randomly and rotated every few days. In addition, each replicate pot was oriented randomly with respect to compass direction.

Pots were first filled with 170-g Turface MVP (Profile, Buffalo Grove, IL), a coarse-grained calcined montmorillonite clay that kept fine-grained media from sifting through drainage holes. On the top of this coarse-grained layer, 400 g of Greens Grade (Profile), a fine-grained (0.25–1.0 mm diameter) calcined montmorillonite clay, was added after being mixed with solid fertilizers as prescribed by treatments detailed below. This clay medium was used because it is easily washed from fine roots and contains only trace amounts of macronutrients.

### Domestication level treatments

We used 12 barley accessions within each domestication level (wild, landrace, cultivar), 36 barley accessions in total (for list of accessions, see Supporting information). In this experiment, accessions represent the unit of replication within domestication level. We acquired seed samples from the United States Department of Agriculture Germplasm Resources Information Network (GRIN) and the Leibniz Institute of Plant Genetics and Crop Plant Research (IPK) in Gatersleben, Germany. We randomly selected 30 accessions from GRIN, which were admitted between 1985 and 2006, and we used six IPK accessions cited in Wacker et al. (2002).

### Fertilizer treatments

Because plants in general, and barley in particular, do not exhibit strong root plasticity in response to potassium (K) (Drew 1975; Hodge 2004), fertilizer levels were determined using N and P nutrient sufficiency (Wild 1988; Troeh and Thompson 2005) and seasonal uptake efficiency for barley (Donahue et al. 1983; Hood 2002). Fertilizer treatment levels were based on an estimated plant dry weight of 5 g comprised of 1% nitrogen (N) and 0.15% phosphorus (P) in a nutrient-sufficient plant. Seasonal uptake efficiencies were assumed to be 50% for N and 20% for P. Solid, slow-release fertilizers (Simplot, Boise, ID) were applied in the forms of polymer-coated potassium nitrate (13-0-43) and triple superphosphate (0-45-0). Low fertilizer level fell short of minimum sufficiency requirements (0.091 g N, 0.019 g P), whereas high fertilizer level exceeded sufficiency requirements (0.46 g N, 0.097 g P).

Fertilizers were either distributed homogeneously throughout the pot (‘whole’) or heterogeneously, such that only one-half of the pot was supplemented with fertilizer (‘split’). To apply these fertilizer distribution treatments, each pot was placed in a round metal miter box and divided vertically into half with a tight-fitting metal partition. A groove in the miter box held the partition in place and later served as a guide for cutting the pots in half. Pot halves were filled individually with soil media mixed according to the assigned fertilizer level. After the pot halves were filled, the metal divider was removed so that roots could grow into both sides of the pot.

Barley plants were grown in a two-way factorial experiment in which two fertilizer level treatments (low versus high) were crossed with two fertilizer distribution treatments (whole versus split pot). Each of the 36 accessions described above was planted in each of the resulting four soil treatment combinations (i.e., there was a single replicate of each accession within each soil treatment combination). Of the 144 seeds that were planted (one seed per pot), 134 germinated and survived.

### Plant harvest and root scanning

After shoots were harvested, pots were placed in the metal miter box and sawed in half along the same axis that divided the pots when they were filled. Each half of the root system was washed separately and stored frozen before scanning. To compare root growth between sides of the pot, a correction coefficient was used to calculate the exact volume of each side of the pot.

Roots were scanned following the protocols detailed in Bouma et al. (2000). Specifically, roots were washed and stained with excess neutral red (0.5-g solid neutral red dye L^−1^ deionized water) for 24 h before being rinsed and stored frozen. Individual samples were thawed, suspended in water in a 20 cm × 30 cm plexiglass tray, scanned at 400 dpi with an Epson 1680 flatbed scanner, and analyzed using WinRHIZO™ (Regent Instruments, Quebec, Canada) with automatic threshold based on gray levels. WinRHIZO™ uses a ‘skeletonization’ method to estimate root parameters (e.g., length, width, diameter) by converting grayscale images into binary data (Himmelbauer et al. 2004). Root and shoot samples were oven-dried (65°C, 24 h) and weighed.

We measured shoot weight, average root diameter, as well as root weight, length, volume, and area. We also calculated several parameters to characterize root systems, including root length density (RLD; km root m^−3^ soil), specific root length (SRL; km root kg^−1^ root), root weight ratio (RWR; g root mass g^−1^ total plant mass), and differential allocation of root weight between sides of split pots (g roots on fertilized side of split pot g^−1^ total roots). Correlations among root weight, root length, root volume, and root surface area were all highly significant (*P* < 0.0001, *r* ≥ 0.9, *n* = 134). Therefore, our calculations of differential root allocation between sides of split pots, which measured differences in root weight, would yield qualitatively similar results if they incorporated differences in root length, volume, or area instead.

Root length density reflects total root growth in the pot volume and is increased by increased branching in the root system. Specific root length is highly correlated with root diameter and surface area, with higher values indicating lower cost roots. Root weight ratio indicates the proportion of a plant’s biomass that is allocated to its root system. Differential root allocation is the proportion of the root system that grew on the fertilized side of split pots and is an indication of within-plant plasticity.

### Statistical analyses

We used sas statistical software v. 9.2 (SAS Institute, Cary, NC), procedure statement ‘PROC CORR,’ to perform two sets of Pearson correlations. First, we correlated root weight, root length, root volume, and root area to evaluate whether those variables would yield qualitatively similar results in subsequent analyses. Those parameters are typically similar indications of root system size, and we sought to limit comparisons involving qualitatively interchangeable character traits. Second, we correlated differential root allocation (log-transformed) with total plant weight (log-transformed) for the split fertilizer distribution treatment to test whether root plasticity affected plant fitness at each domestication level and each fertilizer level.

We also conducted a mixed-model analysis of covariance (ancova in SAS PROC MIXED) on plant weight, RWR, RLD, SRL, average root diameter, and differential allocation. Accession, nested within domestication level, was treated as a random effect. Domestication level, fertilizer level, fertilizer distribution, and their interactions were treated as fixed effects. Accession age (i.e., number of years since the accession was submitted to the germplasm repository) and standardized seed weight were used as covariates for each ancova. We used a Bonferroni correction to account for multiple ancova comparisons and to establish an experiment-wise error rate of α = 0.05. Owing to incomplete germination and the resulting unbalanced design, least squares means (LS-means) are reported.

Accession age was a helpful covariate because the effective population size of an accession, and hence its genetic variation, decreases each time it is grown out to rejuvenate the germplasm in the repository (Parzies et al. 2000). Standardized seed weight was the difference between the weight of an individual seed and the average seed weight for that domestication level. It was important to standardize seed weight in this way because significant differences in seed weight among domestication levels were primarily because of the large glumes in wild barley. Levene and Shapiro–Wilk tests were used to evaluate the assumptions of normality and homogeneity of variance, respectively. Parameters that did not meet the assumption of homogeneity of variance were weighted with the inverse of the residual variance (Welch 1951).

## Results

**Figure 1:**
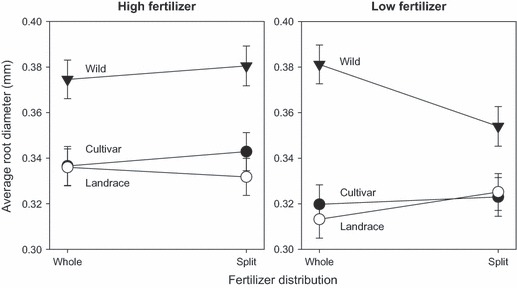
Three-way interaction between domestication level, fertilizer level, and fertilizer distribution for average root diameter (*P* = 0.0095). Values are LS-means ± SE from mixed-model ancova.

A significant three-way interaction among domestication level, fertilizer level, and fertilizer distribution for average root diameter (Fig. 1; *P* = 0.0095) indicated that the expression of within-plant plasticity for average root diameter occurred primarily in wild barley and was most pronounced under low nutrient levels.

Plant size and root plasticity were positively correlated with barley, especially in domesticated cultivars. This was indicated by a significant, overall positive correlation between differential allocation and total plant weight for pots with split fertilizer distribution for barley (*P* = 0.02, *r* = 0.29, *n* = 67) and for cultivars in particular (*P* = 0.02, *r* = 0.49, *n* = 23). In addition, differential root allocation and total plant weight were significantly correlated at both high and low fertilizer levels (*P* = 0.02, *r* = 0.39, *n* = 34 and *P* = 0.01, *r* = 0.44, *n* = 33, respectively). However, the only domestication level by fertilizer level combination that exhibited a significant correlation between differential root allocation and total plant weight was cultivars in low fertilizer pots (*P* = 0.001, *r* = 0.86, *n* = 11).

**Figure 2:**
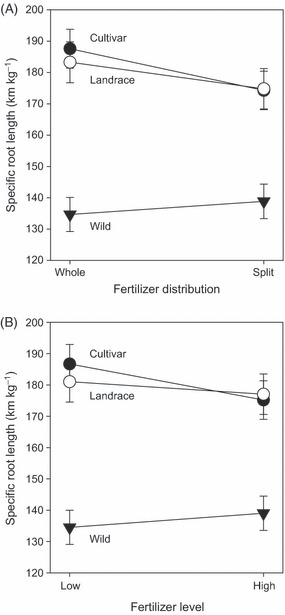
(A) Interaction between barley domestication level and fertilizer distribution for specific root length (SRL) (*P* = 0.0092). Values are LS-means ± SE from mixed-model ancova. (B) Interaction between barley domestication level and fertilizer level for SRL (*P* = 0.0249). Nonsignificant trend after Bonferroni correction. Values are LS-means ± SE from mixed-model ancova.

Wild barley increased SRL, whereas domesticated barley decreased SRL, in response to heterogeneous soils (i.e., split fertilizer distribution), as indicated by a significant interaction between domestication level and fertilizer distribution for SRL (Fig. 2; *P* = 0.0092). As suggested by the interaction between domestication and fertilizer level for SRL (Fig. 2; *P* = 0.0249), wild barley increased SRL in high fertilizer pots, while domesticated barley exhibited a decrease in SRL. However, this interaction became nonsignificant after Bonferroni correction.

Total plant weight was significantly greater for barley plants grown in high fertilizer pots across all domestication levels and fertilizer distribution treatments (Table 1; *P* < 0.0001). Plant weight was significantly lower for wild barley compared with cultivars and landraces across all treatments (Table 2; *P* = 0.0032). Root length density and SRL were also significantly lower in wild barley compared with landraces and cultivars (Table 2; *P* < 0.0001 and *P* < 0.0001, respectively).

**Table 1:** Effects of fertilizer level on barley root systems

			Fertilizer level	
			
Variable	Unit	*P*	Low	High
Total plant weight	g	<0.0001	0.56 ± 0.03	0.76 ± 0.03
Root weight ratio		<0.0001	0.34 ± 0.006	0.27 ± 0.006

Values are LS-means ± SE.

**Table 2:** Differences in root systems among barley domestication levels

			Domestication level		
			
Variable	Unit	*P*	Wild	Landrace	Cultivar
Total plant weight	g	0.0032	0.57 ± 0.03	0.74 ± 0.04	0.67 ± 0.04
Root length density	km m^−3^	<0.0001	25.3 ± 2	43.6 ± 3	39.2 ± 3
Specific root length	km kg^−1^	<0.0001	137 ± 5	179 ± 6	181 ± 6

Values are LS-means ± SE.

Root weight ratio was greater in low fertilizer pots across all domestication levels and fertilizer distribution treatments, indicating that barley plants in low-nutrient soils invested more resources in their root systems (Table 1; *P* < 0.0001). In addition, RWR was slightly greater in pots with split fertilizer distribution, suggesting that barley plants may have developed larger root systems when grown in heterogeneous soils, regardless of fertilizer level (Table 3, *P* = 0.0165, nonsignificant after Bonferroni correction). Differential root allocation between sides of split fertilizer pots demonstrated that barley plants allocated more root weight to the fertilized side of split pots (Table 3; *P* = 0.0002).

**Table 3:** Effects of fertilizer distribution on barley root systems

		Fertilizer Distribution	
		
Variable	*P*	Whole	Split
Root weight ratio*	0.0165	0.30 ± 0.006	0.32 ± 0.006
Differential root allocation	0.0002	0.50 ± 0.01	0.57 ± 0.01

* Nonsignificant after Bonferroni correction.

Values are LS-means ± SE.

## Discussion

Overall, root diameter was larger in high fertilizer pots, and wild barley exhibited larger root diameter than domesticated barley. However, the three-way interaction between domestication, fertilizer level, and fertilizer distribution for average root diameter (Fig. 1) demonstrates that wild barley exhibited greater within-plant plasticity for this trait and that this plasticity was most pronounced under low nutrient levels. Therefore, wild barley appears to be more responsive to fine-grained soil heterogeneity (i.e., split pots) under low-nutrient conditions – presumably similar to their natural environment – whereas domesticated barley expresses greater plasticity in response to coarse-grained heterogeneity at the among-plant level (i.e., whole pots at different fertilizer levels).

Wild barley’s expression of within-plant plasticity in low-nutrient soils might suggest that nutrient distribution is patchy at smaller scales in the wild compared with cultivated lands. This would be consistent with research by Paz-Gonzalez et al. (2000), where sampling at two-meter intervals demonstrated that soil nutrient distribution is relatively more heterogeneous on wild lands compared with cultivated lands. Additional support is provided by Bennett et al. (2005) who showed that wild lands (i.e., prairies) are relatively more variable in soil P at smaller spatial scales (i.e., 1- to 2-m intervals) than human-dominated landscapes.

Soil nutrient distributions on agricultural and wild lands have not been examined extensively at the submeter level, arguably the most appropriate scale for cereal grain root systems, but studies have examined nutrient distribution separately in natural and agricultural landscapes. For example, inorganic nitrogen distribution in the topsoil of a 60 m × 10 m agricultural plot (i.e., top 15 cm) was found to be relatively homogeneous, though spatial variation increased in deeper soils and nitrate showed greater spatial variation than ammonium (Jackson and Bloom 1990). In contrast, significant amounts of variation in nutrient distribution were measured in 0.25-m^2^ areas of natural sagebrush-steppe habitat (Jackson and Caldwell 1993).

Total plant weight has been shown to be a good proxy for overall reproductive fitness in barley (de Wit 1960). The correlation between total plant weight and differential root allocation was significant across all levels of barley domestication. This correlation between root plasticity and fitness appears to have been driven largely by cultivars, the only domestication level that individually exhibited the same correlation. In addition, the benefits of within-plant root plasticity in barley cultivars were only significant under low-nutrient conditions. It may be that in high nutrient levels, cultivars are nutrient sufficient and do not obviously benefit from root plasticity. This indication that the fitness of barley in general, and modern cultivars in particular, is affected by root plasticity is of interest because wild barley appears to harbor the greatest capacity for within-plant plasticity (Fig. 1). However, this higher within-plant plasticity in wild barley did not appear to translate into greater plant weight, as found for cultivars. We realize that this lack of correlation should be interpreted with caution because it is possible that landraces and wild forms of barley were nutrient sufficient in the low fertilizer treatment employed in this experiment. For landraces and wild barley, even lower nutrient levels may be required to reveal the correlation between plant size and root plasticity that we observed in cultivars.

As expected, barley plants grew larger in pots with high fertilizer level. While relative growth rate can affect measurements of root foraging (Aanderud et al. 2003), RWR accounted for differences in relative growth rate by including total plant weight in the denominator of the response variable. In high fertilizer pots, barley plants decreased biomass allocations to roots from 34% to 27% of total plant mass (Table 1). This main effect of fertilizer level, consistent with other studies that generally report decreased RWR in response to high nutrient levels, indicates that barley plants exhibit among-plant plasticity in response to nutrient availability (Reynolds and D’Antonio 1996; Bonifas et al. 2005; Osone and Tateno 2005; McCarthy and Enquist 2007).

Barley plants also tended to allocate more biomass to their root systems when the fertilizer treatment was distributed heterogeneously (Table 3). The literature reports a range of RWR responses to nutrient distribution (Maestre and Reynolds 2007; Ma and Rengel 2008), which may reflect variation in the absorptive condition of roots in the different studies (Reynolds and D’Antonio 1996). In our experiment, plants were harvested at a relatively young age, so it is assumed that measures of root weight were correlated with absorptive capacity. We think this is a reasonable assumption because root systems in young plants are comprised of new, actively growing roots with high-absorptive capacity, while mature plant root systems may contain a significant number of dead or senescent roots. This assumption is further supported by our observations that RWR was responsive to changes in fertilizer level and fertilizer distribution.

At the among-plant scale of root plasticity, barley plants responded to low fertilizer treatment with an increased overall allocation to root growth. In contrast, at the within-plant level of root plasticity, we observed that barley plants allocated a greater proportion of their root system to the fertilized side of split pots (Table 3, differential root allocation). This result, consistent with other studies (Forde 2002; Wang et al. 2006), indicates that root:shoot allocation is affected by the scale of the nutrient patch relative to the root system and is evidence of local root foraging at the within-plant scale.

Mycorrhizae and their influence on nutrient capture in plants (Hodge 2006) may help explain our observations of thicker roots and smaller SRL in wild barley compared with domesticated barley. Fine roots facilitate nutrient uptake in low-nutrient environments, but studies have shown that mycorrhizae can functionally substitute for root hairs in P uptake (Jakobsen et al. 2005) and that mycorrhizal dependency can be negatively correlated with phosphate uptake ability (Tawaraya 2003). One explanation for the larger root diameter we observed in wild barley is that these wild forms may rely more heavily on mycorrhizal associates for nutrient uptake than either landraces or cultivars, and sufficient numbers of those mycorrhizae were not present in our containerized experiment.

Therefore, our observation that wild barley plants were smaller than landraces and cultivars, as indicated by total plant weight and RLD, may have been influenced by a lack of mycorrhizal associates in this experimental design. Future research in barley root plasticity might consider the role of mycorrhizal associations in nutrient acquisition among the different domestication levels and whether wild barley plants are more dependent on mycorrhizal associations than old landraces and modern cultivars.

Our study suggests that root plasticity in barley operates at two different scales: among plants (e.g., RWR decreased in response to increased fertilizer levels) and within plants (e.g., differential root allocation within split pots). These results are a robust comparison of root plasticity among barley domestication levels because accession samples were selected at random with respect to origin, and the main effects of fertilizer level and fertilizer distribution include variation from three distinct levels of domestication. Because dry matter fractionation in cereal crops is conservative across the levels of domestication (Wacker et al. 2002), there may be potential to incorporate traits for greater root plasticity from wild barley into cultivar accessions that can translate such plasticity into increased yield on infertile and heterogeneous soils.

## Data archiving statement

Data deposited in the Dryad repository: doi:10.5061/dryad.b7093hv2.

## Supporting Information

**Table S1**. Barley accessions used to examineroot plasticity among three domestication levels: wild form (wild,*Hordeum spontaneum*), old landraces (landrace, *Hordeumvulgare*), and modern cultivars (cultivar,*H. vulgare*).
