# Supplementary material for: Evolution of root plasticity responses to variation in soil nutrient distribution and concentration
Source: Evol Appl. 2012 Dec;5(8):850–7. doi: 10.1111/j.1752-4571.2012.00263.x (PMC3552402; doi:10.1111/j.1752-4571.2012.00263.x)
Supplement: Supplementary file 1 [file eva0005-0850-SD1.docx]

# Supplementary Materials (accession list)

Barley accessions used to examine root plasticity among three domestication levels: wild form (wild, *Hordeum spontaneum*), old landraces (landrace, *H. vulgare*), and modern cultivars (cultivar, *H. vulgare*).

| Domestication Level | Accession | Source |
| --- | --- | --- |
| Wild | PI 560559* | Siirt, Turkey |
| Wild | PI 559556* | Urfa, Turkey |
| Wild | PI 554428* | Kars, Turkey |
| Wild | PI 554426* | Hakkari, Turkey |
| Wild | PI 554425* | Izmir, Turkey |
| Wild | PI 531859* | Jerusalem, Israel |
| Wild | PI 531858* | Northern Israel |
| Wild | PI 531855* | Northern, Israel |
| Wild | PI 531852* | Central, Israel |
| Wild | PI 531851* | Southern, Israel |
| Wild | HOR 2701† | Turkmenistan |
| Wild | HOR 2700† | Turkmenistan |
| Landrace | PI 606312* | Saudi Arabia |
| Landrace | PI 584975* | Hebei, China |
| Landrace | PI 573907* | Mahakali, Nepal |
| Landrace | PI 573852* | Sagarmatha, Nepal |
| Landrace | PI 573848* | Sagarmatha, Nepal |
| Landrace | PI 564655* | Jiangsu, China |
| Landrace | PI 564631* | Baluchistan, Pakistan |
| Landrace | PI 559507* | Nepal |
| Landrace | PI 510568* | Puno, Peru |
| Landrace | PI 510559* | Puno, Peru |
| Landrace | HOR 1842† | Afghanistan |
| Landrace | HOR 11312† | Albania |
| Cultivar | PI 610232* | Xizang, China |
| Cultivar | PI 599632* | Slovakia |
| Cultivar | PI 592282* | England, United Kingdom |
| Cultivar | PI 592241* | England, United Kingdom |
| Cultivar | PI 592229* | England, United Kingdom |
| Cultivar | PI 573627* | Poltava, Ukraine |
| Cultivar | PI 572591* | Shaanxi, China |
| Cultivar | PI 564721* | Switzerland |
| Cultivar | PI 5479028 | Kentucky, United States |
| Cultivar | PI 525200* | Puy-de-Dome, France |
| Cultivar | HOR 11793† | France |
| Cultivar | HOR 11095† | Korea |

* Accessions provided by the United States Department of Agriculture (USDA) National Genetic Resources Program (NGRP).

† Accessions provided by the Leibniz Institute of Plant Genetics and Crop Plant Research (IPK) in Gatersleben, Germany.
